# Water, Sanitation and Hygiene (WASH) and environmental risk factors for soil-transmitted helminth intensity of infection in Timor-Leste, using real time PCR

**DOI:** 10.1371/journal.pntd.0005393

**Published:** 2017-03-27

**Authors:** Suzy J. Campbell, Susana V. Nery, Rebecca Wardell, Catherine A. D’Este, Darren J. Gray, James S. McCarthy, Rebecca J. Traub, Ross M. Andrews, Stacey Llewellyn, Andrew J. Vallely, Gail M. Williams, Archie C. A. Clements

**Affiliations:** 1 Research School of Population Health, College of Medicine, Biology and Environment, The Australian National University, Acton, Australian Capital Territory, Australia; 2 Molecular Parasitology Laboratory, QIMR Berghofer Medical Research Institute, Brisbane, Queensland, Australia; 3 School of Public Health, University of Queensland, Brisbane, Queensland, Australia; 4 Clinical Tropical Medicine Laboratory, QIMR Berghofer Medical Research Institute, Brisbane, Queensland, Australia; 5 Faculty of Veterinary and Agricultural Science, The University of Melbourne, Parkville, Victoria, Australia; 6 Menzies School of Health Research, Charles Darwin University, Casuarina, Northern Territory, Australia; 7 Kirby Institute, University of New South Wales, Wallace Wurth Building, Kensington, New South Wales, Australia; George Washington University, UNITED STATES

## Abstract

**Background:**

No investigations have been undertaken of risk factors for intensity of soil-transmitted helminth (STH) infection in Timor-Leste. This study provides the first analysis of risk factors for intensity of STH infection, as determined by quantitative PCR (qPCR), examining a broad range of water, sanitation and hygiene (WASH) and environmental factors, among communities in Manufahi District, Timor-Leste.

**Methods:**

A baseline cross-sectional survey of 18 communities was undertaken as part of a cluster randomised controlled trial, with additional identically-collected data from six other communities. qPCR was used to assess STH infection from stool samples, and questionnaires administered to collect WASH, demographic, and socioeconomic data. Environmental information was obtained from open-access sources and linked to infection outcomes. Mixed-effects multinomial logistic regression was undertaken to assess risk factors for intensity of *Necator americanus* and *Ascaris* infection.

**Results:**

2152 participants provided stool and questionnaire information for this analysis. In adjusted models incorporating WASH, demographic and environmental variables, environmental variables were generally associated with infection intensity for both *N*. *americanus* and *Ascaris* spp. Precipitation (in centimetres) was associated with increased risk of moderate-intensity (adjusted relative risk [ARR] 6.1; 95% confidence interval [CI] 1.9–19.3) and heavy-intensity (ARR 6.6; 95% CI 3.1–14.1) *N*. *americanus* infection, as was sandy-loam soil around households (moderate-intensity ARR 2.1; 95% CI 1.0–4.3; heavy-intensity ARR 2.7; 95% CI 1.6–4.5; compared to no infection). For *Ascaris*, alkaline soil around the household was associated with reduced risk of moderate-intensity infection (ARR 0.21; 95% CI 0.09–0.51), and heavy-intensity infection (ARR 0.04; 95% CI 0.01–0.25). Few WASH risk factors were significant.

**Conclusion:**

In this high-prevalence setting, strong risk associations with environmental factors indicate that anthelmintic treatment alone will be insufficient to interrupt STH transmission, as conditions are favourable for ongoing environmental transmission. Integrated STH control strategies should be explored as a priority.

## Author summary

We present a detailed analysis of WASH, environmental and demographic factors associated with intensity of STH infection in Manufahi District, Timor-Leste, using qPCR. Investigation of risk factors for intensity of STH infection is rarely undertaken, and prior analyses have used microscopic-based eggs per gram of faeces (epg) measures, which are of lower diagnostic accuracy than qPCR. Additionally, few analyses have investigated combined WASH and environmental risk factors in association with STH. This is important due to the extensive potential interrelatedness of environmental, social, behavioural and host factors in any given setting influencing STH survival and transmission. This analysis uses categorical intensity of infection variables for *Necator americanus* and *Ascaris* spp., and advanced statistical modelling to adjust for multinomial intensity outcomes, dependency of observations, effects of poverty, and confounding from other measured variables. As such, this analysis provides a comprehensive assessment of risk factors for STH in Manufahi District, Timor-Leste. This is of importance for development of policy and programmatic decisions; risk factors need to be considered not only for their clinical and statistical significance, but more broadly in terms of what may represent modifiable pathways for STH transmission.

## Introduction

Surprisingly little evidence convincingly demonstrates the benefits of water, sanitation and hygiene (WASH) interventions on reducing soil-transmitted helminth (STH) infections [1,2]. Yet it is widely believed that WASH improvements together with anthelmintics could break STH transmission cycles in settings in which anthelmintics alone are insufficient [3,4]. There has been inadequate epidemiological investigation of the role of improved WASH in reducing the STH burden, but there is a growing need for evidence to enable more effective investment in WASH and integrated strategies for STH control.

Intensity of STH infection is important to assess in epidemiological analyses. STH are highly aggregated in humans, with a small number of people harbouring large numbers of helminths, and the majority harbouring few or none [5]. As with prevalence, intensity of worm burden is marked within various groups of the community such as different age groups and gender [6]. This well-described phenomenon is a key feature of this macroparasite relationship with the human host. For quantitative investigations it is therefore problematic to use solely prevalence of infection as an indicator of STH burden or transmission, because large changes in intensity may only be accompanied by small changes in prevalence [6]. STH do not reproduce within the host; infection intensity depends on the time and extent of exposure [7]. Where STH are endemic, maximum worm intensity usually occurs at ages five to ten for *Ascaris lumbricoides* and *Trichuris trichiura*, and in adolescence or early adulthood for hookworm [6]. Whilst the reasons for this are unknown, it may be due to behavioural and social factors, nutritional status, genetic and immunological factors [5,8–11]. There is evidence that some individuals are predisposed to heavy or light STH infections [9,12]. Intensity of *T*. *trichiura* infection reacquired by an individual after treatment has been found to be significantly correlated with the intensity of infection prior to treatment [13]. Additionally, intensity of infection with STH has been identified as substantially greater when any of the species occurred in combination with one or more of the others [14], probably also due to exposure, genetic and immunological factors, which could then act in determining risk of associated morbidities. Despite this knowledge, there is much focus on the use of prevalence to measure STH infection endemicity.

The relationships between intensity of STH infection and risk factors have been inadequately explored, yet could provide useful information as to why intensities differ by host age, environment, and helminth species. Because a key feature of the STH life cycle is the soil-dwelling stage, STH survival, development and transmission potential all rely on a complex assortment of environmental, social, behavioural and host factors. Therefore, in addition to investigating associations between WASH and STH, community-based associations must be considered within their environmental context [15,16]. Although more evidence is required, STH associations with WASH have been systematically appraised [2]. Studies have additionally identified temperature, rainfall, soil porosity and pH, vegetation and elevation ranges as influencing *N*. *americanus* larval development and STH transmission [16,17]. We have previously separately reported on WASH [18] and environmental [19] risk factors for STH prevalence in Manufahi District, Timor-Leste. Given exposure-related risks, and associations between heavy-intensity infection and morbidity, this analysis was conducted to investigate whether WASH- and environmental-related risk factors in this district may also be associated with infection intensity, using categories derived from quantitative PCR (qPCR), a highly sensitive and specific diagnostic technique [20]. By combining data on both WASH and environmental risk factors this analysis provides a more complete picture of risks and thereby augments the current knowledge of risk factors for STH in Timor-Leste. Knowledge of WASH risk factors will be used to inform control strategies in this country. Whilst many environmental risk factors may not be modifiable, the inclusion of these factors will enable targeting of control strategies to areas of greatest need. This is one of very few extensive investigations of combined WASH and environmental risk factors for STH undertaken. It is additionally the first epidemiological analysis of risk factors undertaken using categorised intensity of STH infection from qPCR.

## Results

**Fig 1:**
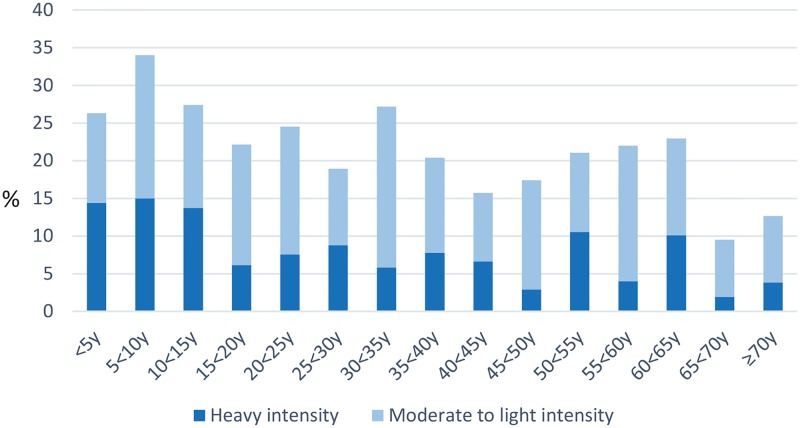
Intensity of *Ascaris* infection by age group.

**Fig 2:**
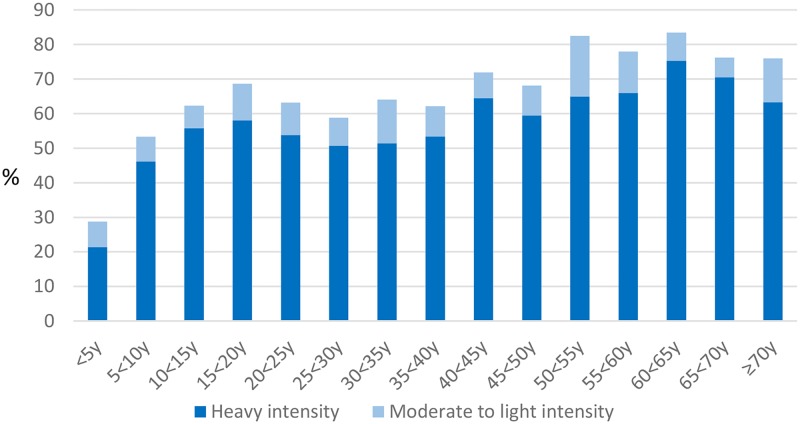
Intensity of *N*. *americanus* infection by age group.

From 24 communities, 2827 eligible people provided baseline survey data, of whom 2152 participants (1038 males, 1114 females) completed both an individual questionnaire and provided a stool sample and were included in this analysis (Table 1, [18]). Using our infection intensity cut-points, more than half (52%) of participants had heavy-intensity *N*, *americanus* infection; 10% had heavy-intensity *Ascaris* infection (Table 1). There was very low prevalence of water or sanitation infrastructure, and most households owned few assets. Most heavy-intensity *Ascaris* infection occurred in children (Fig 1). Heavy-intensity *N*. *americanus* infections were more spread across age groups (Fig 2). Heavy-intensity *Ascaris* infection varied significantly by socioeconomic quintile (*P* = 0.012); *N*. *americanus* infection intensity did not (*P* = 0.468).

**Table 1:** Selected baseline characteristics of study participants (*N* = 2152)^a^.

Characteristic	n (%)
*N*. *americanus* heavy-intensity infection	1,117 (52)
*N*. *americanus* moderate- to light-intensity infection	182 (8.5)
*Ascaris* spp. heavy-intensity infection	217 (10)
*Ascaris* spp. moderate- to light-intensity infection	311 (15)
*Ancylostoma* spp. prevalence	102 (4.7)
No STH infection	665 (31)
*G*. *duodenalis* prevalence	268 (13)
Male sex	1,038 (48)
Improved household water source	106 (18)
Uses unhygienic toilet	1727 (80)
Uses soap/ash to wash hands	1625 (76)
Always wears shoes when toileting	1361 (63)
Never attended school	451 (44)
Not finished primary school	207 (20)
Completed primary but not secondary school	245 (24)
Completed secondary school or higher	118 (12)
Reported taking anthelmintic in previous 12 months	97 (4.5)
Acidic soil (pH 5.5–6.5)	686 (32)
Neutral soil (pH 6.5–7.3)	986 (46)
Alkaline soil (pH 7.3–8.4)	480 (22)
Sandy-loam soil type	611 (28)
Woody savanna (*Ascaris* model)	661 (31)
Woody savanna and evergreen forest (*N*. *americanus* model)	739 (34)
	**Median (interquartile range)**
Mean temperature (°C) in coldest quarter (June-August) (*Ascaris* model)	23 (20.5, 24.7)
Temperature range (°C; maximum temperature in the hottest month—minimum temperature in coldest month) (*N*. *americanus* model)	11.4 (11.2, 11.5)
Mean precipitation (cm) in wettest quarter (December-February) (*Ascaris* model)	29.1 (23.1, 35.3)
Mean precipitation (cm) in driest month (September) (*N*. *americanus* model)	1.7 (1.4, 2.0)
Slope (°)	14.1 (3.5, 18.3)
Elevation per 100m	4.2 (1.3, 7.4)
NDVI (average)	75.1 (70.4, 77.7)

**Notes:** ^**a**^Baseline WASH and environmental risk factors for this population have previously been reported [18, 19]. Parasitological outcomes determined by PCR, types of household latrines observed by interviewer, remaining WASH data are self-reported. Household water source reported at household (*N* = 594) not individual level. Education level asked of adults only (*N* = 1090). “Improved” household water source as defined by WHO/ UNICEF Joint Monitoring Programme (JMP) for Water Supply and Sanitation to include piped water into dwelling or yard, public tap or standpipe, tubewell or borehole, protected dug well, protected spring [33]. “Unhygienic toilet” defined as any people who did not use a hygienic toilet (this included people who used a mixture of hygienic and non-hygienic toilets; hygienic toilets defined as use of a house/school/village/neighbour toilet and nothing else). “Always wearing shoes” was contrasted to sometimes/never wearing shoes.

### Factors associated with *N*. *americanus* intensity of infection

Environmental factors were associated with *N*. *americanus* infection (Table 2). Of particular note, precipitation, measured in centimetres, was significantly associated with a six-fold increased risk of moderate-intensity (adjusted risk ratio [ARR] 6.1, 95% confidence interval (CI) 1.9, 19.3), and seven-fold increased risk of heavy-intensity infection (ARR 6.6, 95%CI 3.1, 14.1), compared to no infection. Sandy-loam soil around the house was associated with more than two-fold higher risk of moderate-intensity (ARR 2.1, 95%CI 1.0, 4.3), and heavy-intensity infection (ARR 2.7, 95%CI 1.6, 4.5), respectively, compared to other soil types. Increasing elevation above sea-level was associated with slightly reduced risk of heavy-intensity infection (ARR 0.90, 95%CI 0.83, 0.97), but was not associated with moderate-intensity infection (ARR 0.94, 95%CI 0.83, 1.1). Increasing normalised difference vegetation index (NDVI) was associated with increased risk for heavy-intensity infection (ARR 1.1, 95%CI 1.0, 1.1). Soil acidity was not included in *N*. *americanus* regression models (*P*>0.2 on univariable analysis).

**Table 2:** Relative risk ratios for intensity of *N*. *americanus* infection, Manufahi District, Timor-Leste.

Parameter	*N*. *americanus* moderate-intensity *N* = 191					*N*. *americanus* heavy-intensity *N* = 1155				
	RR	95% CI	ARR	95% CI	*P*	RR	95% CI	ARR	95% CI	*P*
**Domain: General**										
Age in years	1.0***	1.0, 1.0	N/A			1.0***	1.0, 1.0	N/A		
Age group 6 to 11 years^b^	**1.5**	0.88, 2.7	1.2	0.55, 2.8	0.621	4.6***	3.1, 6.6	3.2	1.8, 5.8	<0.0001
Age group 12 to 17 years^b^	2.2**	1.1, 4.2	1.6	0.63, 4.2	0.311	6.8***	4.3, 10.6	4.8	2.5, 9.3	<0.0001
Age group 18–64 years^b^	3.3***	2.1, 5.3	1.6	0.80, 3.3	0.174	8.1***	5.7, 11.4	4.7	2.8, 7.9	<0.0001
Age group 65+ years^b^	4.8***	2.3, 10.0	4.4	1.6, 11.9	0.004	15.9***	9.3, 27.0	9.6	4.4, 20.7	<0.0001
Male sex: age group 6 to 11 years			1.1	0.34, 3.5	0.891			2.0	0.92, 4.4	0.081
Male sex: age group 12 to 17 years			1.6	0.41, 6.0	0.517			2.3	0.89, 5.7	0.087
Male sex: age group 18–64 years			3.3	1.3, 8.7	0.015			3.6	1.8, 7.3	<0.0001
Male sex: age group 65+ years			0.53	0.11, 2.5	0.419			1.7	0.60, 4.8	0.318
Male sex^c^	1.5**	1.1, 2.1	0.95	0.43, 2.1	0.901	2.7***	2.1, 3.3	1.3	0.73, 2.5	0.351
**Domain: Individual hygiene**										
Sometimes/never wears shoes inside house	**0.74**	0.52, 1.1				0.76**	0.59, 0.98			
Sometimes/never wears shoes outside house	0.86	0.60, 1.2				0.69***	0.53, 0.88			
Sometimes/never wears shoes when toileting	**0.70**	0.48, 1.0				0.64***	0.49, 0.83			
**Domain: Individual sanitation**										
Uses unhygienic toilet	0.90	0.57, 1.4				1.0	0.70, 1.4			
Household has toilet	1.0	0.67, 1.6				0.85	0.60, 1.2			
Cleans self with water and hand only after toileting	**0.66**	0.41, 1.1				0.42***	0.29, 0.60			
Cleans self by other method after toileting	**0.70**	0.45, 1.1				0.61***	0.44, 0.84			
**Domain: Household sanitation**										
Household toilet: Pit latrine without slab	0.77	0.35, 1.7				**1.7**	0.89, 3.1			
Household toilet: Other toilet type	0.87	0.13, 5.9				0.76	0.13, 4.4			
No household toilet/no answer	0.84^a^	0.47, 1.5				1.5^a^	0.92, 2.6			
Toilet observed to be dirty	0.77	0.34, 1.7				1.8**	0.94, 3.5			
Household rubbish disposed of by burning only	0.99	0.64, 1.5				1.0	0.72, 1.5			
Household rubbish disposed of by other method	1.0	0.64, 1.6				0.93	0.65, 1.3			
**Domain: Household water supply**										
Main water supply: piped water to dwelling	^a^		^a^			^a^		^a^		
Main water supply: piped water to yard	**2.1**	0.83, 5.2	1.6	0.58, 4.3	0.373	1.0	0.46, 2.3	1.6	0.53, 2.7	0.718
Main water supply: piped water shared	0.18^a^	0.02, 1.6	0.14^a^	0.02, 1.3	0.083	0.49	0.16, 1.5	0.32	0.12, 0.84	0.021
Main water supply: tubewell/borehole	0.48^a^	0.13, 1.8	1.4^a^	0.42, 4.6	0.583	0.29**	0.11, 0.81	0.72	0.34, 1.5	0.382
Main water supply: unprotected dug well	0.97^a^	0.11, 8.9	1.3^a^	0.13, 13.2	0.822	0.44^a^	0.07, 2.7	0.71^a^	0.10, 5.1	0.737
Main water supply: protected spring	0.68^a^	0.13, 3.6	0.61^a^	0.11, 3.5	0.583	1.2	0.42, 3.7	0.99	0.33, 3.0	0.982
Main water supply: surface water	**1.7**	0.99, 2.7	1.9	1.1, 3.2	0.020	**1.2**	0.79, 1.8	1.3	0.85, 1.9	0.264
Main water supply: in household compound	0.91	0.50, 1.6				0.78	0.47, 1.3			
Distance to main water supply: 15 min or more	1.1	0.75, 1.7				**1.3**	0.93, 1.8			
Main water supply not running at least 1 week/month	0.78^a^	0.34, 1.8				0.98	0.53, 1.8			
Main water supply not running at least 1 month/year	0.77	0.46, 1.3				0.60**	0.39, 0.93			
Water stored in covered container only	1.2	0.78, 1.9				**1.4**	1.0, 2.0			
Household water is boiled	0.60**	0.40, 0.90	0.52	0.34, 0.80	0.003	0.88	0.63, 1.2	0.73	0.52, 1.0	0.057
**Domain: Household hygiene**										
Household has a food garden	0.62	0.34, 1.2				1.3	0.81, 1.9			
Human faeces used on food garden	2.6	0.80, 8.3				1.8	0.61, 5.2			
**Domain: Household socioeconomic**										
1 person aged <5 years in household	0.68**	0.45, 1.0	0.81	0.52, 1.3	0.338	0.39***	0.28, 0.53	0.57	0.40, 0.82	0.002
2 or more people aged <5 years in household	0.43***	0.27, 0.67	0.57	0.34, 0.94	0.028	0.39***	0.28, 0.55	0.78	0.53, 1.2	0.221
1 person aged >65 years in household	1.3	0.63, 2.5				1.0	0.58, 1.9			
2 or more people aged >65 years in household	1.9**	1.1, 3.5				1.7**	1.1, 2.9			
Socioeconomic quintile 4	1.9**	1.1, 3.4	1.4	0.75, 2.5	0.2340	1.8**	1.2, 2.9	1.8	1.2, 2.9	0.0213
Socioeconomic quintile 3	1.5	0.87, 2.7	1.2	0.64, 2.2		1.5	0.97, 2.4	1.8	1.1, 2.9	
Socioeconomic quintile 2	1.3	0.70, 2.2	1.7	0.91, 3.0		1.8**	1.1, 2.8	1.7	1.1, 2.8	
Socioeconomic quintile 1 (poorest)	1.3	0.70, 2.2	2.0	1.1, 3.7		1.6**	1.0, 2.5	2.2	1.3, 3.6	
**Domain: Village**										
Village toilet type: Pit latrine without slab	0.43^a^	0.06, 3.0				0.93	0.17, 5.2			
Village toilet type: Pit latrine with slab	0.23^a^	0.03, 2.2				1.1	0.18, 6.0			
Village rubbish disposed of by burning only	0.55	0.10, 3.1				0.66	0.13, 3.4			
Village rubbish disposed of by other method	0.66	0.31, 1.4				0.80	0.39, 1.6			
**Domain: qPCR**										
*Ascaris* spp. infection	1.2	0.81, 1.9				1.2	0.86, 1.6			
*Ancylostoma* spp. infection	1.4^a^	0.50, 4.0	1.0^a^	0.32, 3.2	0.989	4.0***	2.2, 7.5	4.1	2.1, 8.0	<0.0001
*G*. *duodenalis* infection	0.61**	0.37, 1.0	0.86	0.50, 1.5	0.585	0.44***	0.31, 0.61	0.71	0.49, 1.0	0.076
**Domain: Individual recent history**										
Deworming treatment taken within last 12 months	0.74^a^	0.33, 1.7				0.43***	0.23, 0.81			
3 or more bowel motions in last 24 hours	0.74^a^	0.28, 2.0	0.82^a^	0.30, 2.4	0.711	0.32**	0.15, 0.70	0.40	0.17, 0.96	0.041
Loose stools during last 24 hours	0.94^a^	0.36, 2.5				0.35***	0.16, 0.76			
Diarrhoea during last 2 weeks	0.90	0.48, 1.7				0.89	0.58, 1.4			
Access to anthelmintics	0.37**	0.17, 0.80				0.35***	0.20, 0.60			
**Domain: Environmental**										
Sandy-loam soil	2.0**	1.1, 3.7	2.1	1.0, 4.3	0.038	2.4***	1.4, 4.0	2.7	1.6, 4.5	<0.0001
Woody savanna & evergreen forest landcover	**2.1**	0.95, 4.4				2.3**	1.2, 4.4			
Temperature (°C)	**0.41**	0.13, 1.4	N/A			**0.47**	0.16, 1.4	N/A		
Precipitation (cm)	5.5***	2.4, 12.8	6.1	1.9, 19.3	0.002	5.6***	3.2, 9.9	6.6	3.1, 14.1	<0.0001
Elevation (m)	**1.1**	0.98, 1.2	0.94	0.83, 1.1	0.268	**1.1**	0.98, 1.2	0.90	0.83, 0.97	0.007
Slope (°)	1.1***	1.0, 1.1				1.1***	1.0, 1.1			
NDVI (average per 0.01)	1.1***	1.0, 1.2	1.1	0.98, 1.7	0.146	1.2***	1.1, 1.2	1.1	1.0, 1.1	0.023

**Notes:** No *N*. *americanus* infection is reference category, i.e., moderate- and heavy-intensity infection need to be interpreted relative to this reference. *N*. *americanus* intensity infection defined according to following PCR cycle threshold (Ct) cut-points: heavy-intensity Ct≤24.6, moderate-intensity Ct>24.6<35, no infection Ct≥35 [23]. RR, relative risk; ARR, adjusted relative risk; CI, confidence interval; *P*, Wald test. *** *P*<0.01, ** *P*<0.05 in univariable analysis, ^a^ less than 10 observations in subgroup; result should be interpreted cautiously. RRs in bold had univariable *P*<0.2 and were entered in multivariable regression models; for correct interpretation of this table, if a variable was significant for moderate-intensity but not heavy-intensity, it was still included, therefore on occasion moderate-intensity adjusted RRs are significant when heavy-intensity adjusted RRs are not, and *vice versa*. Multivariable analysis did not include temperature due to collinearity with elevation; this is indicated as N/A = not applicable. Age (categorical), sex and socioeconomic quintile were included in all multivariable regression models as covariates. Age (continuous) is indicated as NA = not applicable for multivariable models. Water supply variables follow JMP definitions [33], with the exception of “piped water” which was grouped due to low observation numbers. Definitions: “Used unhygienic toilet” any people who did not use a hygienic toilet (this included people who used a mixture of hygienic and non-hygienic toilets; hygienic toilets defined as use of a house/school/village/neighbour toilet and nothing else). “Other household toilet type” indicates hanging latrines (low observation numbers). “Household rubbish disposed of by other method” includes disposing it into a bin, a river, burying it or composting it. “Village rubbish disposed of by other method” includes burying it or disposing of it in the river. Reference categories: **General domain:** lowest age group in the stratum (age 1–5 years); female sex; female sex and age group 1 to 5 years. **Individual hygiene domain:** always wears shoes inside house; always wears shoes outside house; always wears shoes when toileting. **Individual sanitation domain:** uses hygienic toilet only; household has no toilet; cleans self with leaves only after toileting. **Household sanitation domain:** household toilet being a pit latrine with slab; household toilet observed (by interviewer) to be clean; household rubbish disposed of in bush only. **Household water supply domain:** main water supply being an unprotected spring; main water supply located separate from household compound; distance to main water supply less than 15 minutes; main water supply always running; household water stored in an uncovered container; household water is not boiled. **Household hygiene domain:** household does not own food garden; human faeces not used on food garden. **Household socioeconomic domain:** no people aged <5 years in household/no answer; no people aged ≥65 years in household/no answer; socioeconomic quintile 5 (wealthiest). **Village domain:** no village toilet; village rubbish disposed of in bushes only. **qPCR domain:** no *Ascaris* infection; no *Ancylostoma* infection; no *G*. *duodenalis* infection. **Individual recent history domain:** no deworming treatment taken within last 12 months; less than 3 bowel motions in last 24 hours; normal stools during last 24 hours; no diarrhoea in last two weeks; no access to anthelmintics. **Environmental domain:** acidic soil pH; other soil types (clay, clay-loam, sandy-clay, variable); other landcover types (cropland/natural vegetation, savanna).

**Interpretation notes:** Because this table has an interaction term, parameterisation and correct interpretation in the adjusted model are as follows: ^b^ the age group main effect is the stated age group (e.g. six to 11 years) relative to age group one to five years in females because females are the reference group (i.e. demonstrating the relationship between age group and *N*. *americanus* infection intensity in females). Similarly, ^c^ the male sex main effect is being male relative to being female in age group one to five years (reference group). Males in age groups six to 11 years and older is relative to males in age group one to five years (because males and older age groups are not the reference groups).

Co-infection with *Ancylostoma* spp. was associated with four-fold higher risk of heavy-intensity *N*. *americanus* infection (ARR 4.1, 95%CI 2.1, 8.0). *G*. *duodenalis* was marginally non-significant for heavy-intensity infection (ARR 0.71, 95%CI 0.49, 1.0). Due to the sex by age interaction term results are reported separately for females and males within age groups. Relative to no *N*. *americanus* infection, a significant gradient of increased risk of heavy *N*. *americanus* infection intensity with increasing age group was evident for females (ARRs increasing from 3.2 to 9.6; see Table 2), however this was less evident for moderate-intensity infection (with the exception of being aged 65 years or older having four-fold increased risk of infection; ARR 4.4, 95%CI 1.6, 11.9). For males, relative to no infection, being aged 18 to 64 years was significantly associated with more than three-fold increased risk of any intensity infection (moderate-intensity ARR 3.3, 95%CI 1.3, 8.7; heavy-intensity ARR 3.6, 95%CI 1.8, 7.3). Sex in participants aged one to five years (i.e. reference group) was not associated with intensity of infection. A gradient of generally increasing risk of moderate- and heavy-intensity infection was also evident with worsening socioeconomic quintile (being significant across most subgroups for heavy-intensity), with people in the poorest quintile having more than twice the risk of infection for both intensity levels (moderate-intensity ARR 2.0, 95%CI 1.1, 3.7; heavy-intensity ARR 2.2, 95%CI 1.3, 3.6).

Few associations were found between WASH variables and STH outcomes in adjusted analyses. Of note is that a shared piped water supply was associated with strongly reduced risk of heavy-intensity infection compared to an unprotected stream (ARR 0.32, 95%CI 0.12, 0.84), and use of surface water was associated with twice the risk of moderate-intensity infection compared to an unprotected stream (ARR 1.9, 95%CI 1.1, 3.2). Boiling household water was associated with half the risk of moderate-intensity *N*. *americanus* infection compared to not boiling water (ARR 0.52, 95%CI 0.34, 0.80). Having one preschool-aged child in the household was protective against heavy-intensity *N*. *americanus* infection (ARR 0.57, 95%CI 0.40, 0.82). For moderate-intensity infection having one preschool-aged child in the house was not significant (ARR 0.81, 95%CI 0.52, 1.3), but having more than one was associated with reduced risk (ARR 0.57, 95%CI 0.34, 0.94). People reporting three or more bowel motions during the previous 24 hours (indicating diarrhoea) was associated with reduced risk of heavy-intensity infection compared to people who reported less than three bowel motions (ARR 0.40, 95%CI 0.17, 0.96). People who reported having access to anthelmintic drugs and people who reported actually taking deworming treatment within the previous 12 months, was not associated with risk of infection in adjusted models, despite these factors being highly significant in univariable analysis for heavy-intensity infection. Methods of post-defecation anal cleansing, and shoe wearing, all of which were highly significant in univariable analyses for heavy-intensity infection, did not emerge as risk factors in adjusted analyses.

### Factors associated with intensity of *Ascaris* infection

Factors significantly associated with *Ascaris* infection were age, and environmental variables, particularly alkaline soil and elevation above sea level (Table 3). Alkaline soil was significantly associated with highly reduced risks of moderate-intensity (ARR 0.21, 95%CI 0.09, 0.51), and heavy-intensity *Ascaris* infection (ARR 0.04, 95%CI 0.01, 0.25, note low numbers) compared to acidic soils. Neutral pH soil showed no association with risk of infection. Increasing elevation was associated with *Ascaris* infection, with observations of a mild gradient of increasing risk with increasing infection intensity (moderate-intensity ARR 1.3, 95%CI 1.2, 1.4; heavy-intensity ARR 1.4, 95%CI 1.2, 1.7). Increasing NDVI was also associated with mildly increased risk of heavy-intensity infection (ARR 1.2, 95%CI 1.1, 1.4). No WASH variables emerged as risk factors for *Ascaris* infection. Increasing age was associated with reducing risk of both moderate and severe infection intensity on a gradient that was significant for many age groups (particularly for heavy-intensity infections). Sex and socioeconomic status were not risk factors for *Ascaris* infection intensity.

**Table 3:** Relative risk ratios for intensity of *Ascaris* spp. infection, Manufahi District, Timor-Leste.

Parameter	*Ascaris* moderate-intensity *N* = 310					*Ascaris* heavy-intensity *N* = 220				
	RR	95% CI	ARR	95% CI	*P*	RR	95% CI	ARR	95% CI	*P*
**Domain: General**										
Age in years	0.99***	0.98, 1.0	N/A			0.97***	0.96, 0.98	N/A		
Age group 6 to 11 years	1.9***	1.2, 3.0	1.9	1.2, 3.1	0.005	2.3***	1.3, 4.1	2.4	1.3, 4.3	0.004
Age group 12 to 17 years	1.1	0.66, 2.0	1.1	0.63, 1.9	0.739	0.97	0.46, 2.0	0.91	0.43, 1.9	0.807
Age group 18–64 years	0.98	0.65, 1.5	0.95	0.62, 1.4	0.795	0.58**	0.33, 1.0	0.56	0.32, 0.98	0.038
Age group 65+ years	**0.52**	0.26, 1.0	0.52	0.26, 1.0	0.059	0.19***^a^	0.06, 0.63	0.20	0.06, 0.66	0.008
Male sex	1.1	0.83, 1.5	1.1	0.83, 1.5	0.478	0.97	0.66, 1.4	0.88	0.59, 1.3	0.537
**Domain: Individual hygiene**										
Washes hands after defecation and at other times	0.83	0.54, 1.3				0.46**	0.25, 0.84			
Washes hands at other times only (but not after defecation)	0.77	0.46, 1.3				**0.51**	0.24, 1.1			
Sometimes/never wears shoes outside house	0.95	0.70, 1.3				1.7**	1.1, 2.6			
Sometimes/never wears shoes when toileting	**0.82**	0.59, 1.1				1.1	0.68, 1.7			
**Domain: Individual sanitation**										
Used unhygienic toilet	1.3	0.79, 2.0				**2.3**	0.91, 4.6			
Household has toilet	0.82	0.54, 1.2				**0.57**	0.27, 1.2			
Cleans self with water and hand only after toileting	0.85	0.53, 1.3				0.88	0.44, 1.8			
Cleans self by other method after toileting	0.92	0.62, 1.4				0.95	0.50, 1.8			
Village has public toilet	0.75	0.34, 1.7				0.19^a^	0.03, 1.1			
**Domain: Household sanitation**										
Household toilet: Pit latrine without slab	1.4	0.64, 2.9				0.72^a^	0.19, 2.8			
Household toilet: Other toilet type	0.54^a^	0.09, 3.2				0.39^a^	0.02, 6.9			
No household toilet/no answer	1.3	0.73, 2.4				1.5	0.56, 3.9			
Toilet observed to be clean	**0.44**	0.17, 1.1				1.0	0.30, 3.4			
Household rubbish disposed of by burning only	0.82	0.55, 1.3				1.2	0.61, 2.3			
Household rubbish disposed of by other method	0.78	0.52, 1.2				0.38**	0.18, 0.77			
**Domain: Household water supply**										
Main water supply: piped water (to any point)	**1.8**	0.83, 3.8				1.4	0.40, 4.9			
Main water supply: tubewell/borehole	0.15^a^	0.01, 3.1				^a^				
Main water supply: protected spring	1.3^a^	0.37, 4.3				^a^				
Main water supply: surface water	1.4	0.83, 2.3				0.98	0.40, 2.4			
Main water supply located in household compound	**1.5**	0.85, 2.6				0.94	0.35, 2.5			
Main water supply not running at least 1 week/month	0.64	0.31, 1.3				0.73	0.21, 2.5			
Main water supply not running at least 1 month/year	0.79	0.48, 1.3				0.78	0.34, 1.8			
Household water is stored	0.61	0.18, 2.0				0.73	0.12, 4.6			
Secondary water source used	0.82	0.55, 1.2				0.70	0.36, 1.4			
Secondary water source is “improved”	0.86	0.30, 2.4				0.72^a^	0.14, 3.7			
Household water is boiled	0.87	0.58, 1.3				0.93	0.46, 1.9			
**Domain: Household hygiene**										
Household has a food garden	0.73	0.43, 1.2				0.76	0.31, 1.8			
Human faeces used on food garden	1.2	0.39, 3.4				1.7	0.28, 9.7			
**Domain: Household socioeconomic**										
1 person aged <5 years in household	1.5**	1.0, 2.2				**1.5**	0.82, 2.9			
2 or more people aged <5 years in household	1.1	0.71, 1.6				1.5	0.76, 2.8			
1 person aged 5–17 years in household	0.82	0.48, 1.4				0.37**	0.14, 0.95			
2 or more people aged 5–17 years in household	**1.4**	0.92, 2.1				1.6	0.84, 3.1			
1 person aged 65+ years in household	0.67	0.32, 1.4				0.49^a^	0.13, 1.8			
2 or more people aged 65+ years in household	0.85	0.48, 1.5				0.65	0.25, 1.7			
Socioeconomic quintile 4	0.76	0.43, 1.3	0.67	0.38, 1.2	0.6461	1.2	0.48, 3.2	0.76	0.26, 2.2	0.7453
Socioeconomic quintile 3	0.81	0.48, 1.4	0.96	0.56, 1.6		0.96	0.38, 2.5	1.0	0.38, 2.7	
Socioeconomic quintile 2	0.84	0.50, 1.4	0.91	0.54, 1.5		0.92	0.35, 2.4	1.2	0.45, 3.1	
Socioeconomic quintile 1 (poorest)	0.59	0.33, 1.1	0.82	0.48, 1.4		0.66	0.23, 1.9	1.5	0.58, 4.0	
**Domain: Village**										
Village rubbish disposed of by burning only	0.10^a^	<0.01, 2.6				0.05^a^	<0.00, 42.7			
Village rubbish disposed of by other method	0.42	0.11, 1.6				0.35	0.02, 5.7			
**Domain: qPCR**										
*N*. *americanus* infection	1.0	0.76, 1.4				**1.4**	0.88, 2.2			
*Ancylostoma* spp. infection	1.1	0.58, 2.2				2.8**	1.3, 6.3			
*G*. *duodenalis* infection	1.2	0.77, 1.8				1.4	0.77, 2.4			
**Domain: Environmental**										
Soil pH: Alkaline	0.09***	0.03, 0.31	0.21	0.09, 0.51	0.001	0.01***^a^	<0.01, 0.09	0.04^a^	0.01, 0.25	0.001
Soil pH: Neutral	0.54***	0.30, 0.96	0.55	0.33, 0.91	0.019	0.34**	0.12, 0.92	0.35	0.14, 0.84	0.019
Sandy-loam soil	**0.60**	0.33, 1.1				0.24***	0.09, 0.64			
Woody savanna landcover	0.44**	0.23, 0.81				0.28**	0.09, 0.85			
Slope (°)	1.2***	1.1, 1.3				1.4***	1.2, 1.5			
Temperature (°C)	0.61***	0.52, 0.71	N/A			0.42***	0.31, 0.57	N/A		
Precipitation (cm)	1.3***	1.2 1.4				1.5***	1.3, 1.7			
Elevation (m)	1.4***	1.3, 1.6	1.3	1.2, 1.4	<0.0001	1.8***	1.5, 2.2	1.4	1.2, 1.7	<0.0001
NDVI (average per 0.01)	1.1**	1.0, 1.3	1.1	0.97, 1.1	0.201	1.4***	1.1, 1.7	1.2	1.1, 1.4	0.028

**Notes:** No *Ascaris* infection is reference category, i.e., moderate- and heavy-intensity infection need to be interpreted back to this reference. *Ascaris* intensity of infection defined according to following PCR cycle threshold (Ct) cut-points: heavy-intensity Ct≤15.4, moderate-intensity Ct>15.4<31, no infection Ct≥31 [23]. RR, relative risk; ARR, adjusted relative risk; CI, confidence interval; *P*, Wald test. *** *P*<0.01, ** *P*<0.05 in univariable analysis, ^a^ less than 10 observations in subgroup; result should be interpreted cautiously. RRs in bold had univariable *P*<0.2 and were entered in multivariable regression models; for correct interpretation of this table, if a variable was significant for moderate-intensity but not heavy-intensity, it was still included, therefore on occasion moderate-intensity adjusted RRs are significant when heavy-intensity adjusted RRs are not, and *vice versa*. Multivariable analysis did not include temperature due to collinearity with elevation; this is indicated as N/A = not applicable. Age (categorical), sex and socioeconomic quintile were included in all multivariable regression models as covariates. Age (continuous) is indicated as NA = not applicable for multivariable models. No interaction term was required in *Ascaris* models. Water supply variables follow JMP definitions [33], with the exception of “piped water” which was grouped due to low observation numbers. Definitions: “Used unhygienic toilet” any people who did not use a hygienic toilet (this included people who used a mixture of hygienic and non-hygienic toilets; hygienic toilets defined as use of a house/school/village/neighbour toilet and nothing else). “Other household toilet type” indicates hanging latrines (low observation numbers). “Household rubbish disposed of by other method” includes disposing it into a bin, a river, burying it or composting it. “Village rubbish disposed of by other method” includes burying it or disposing of it in the river. Reference categories: **General domain:** lowest age group in the stratum (age 1–5 years); female sex. **Individual hygiene domain:** uses soap/ash to wash hands; washes hands after defecation only; always wears shoes outside house; always wears shoes when toileting. **Individual sanitation domain:** uses hygienic toilet only; household has no toilet; cleans self with leaves only after toileting; village has no public toilet. **Household sanitation domain:** household toilet being a pit latrine with slab; household toilet observed (by interviewer) to be dirty; household rubbish disposed of in bush only. **Household water supply domain:** main water supply being an unprotected spring; main water supply located separate from household compound; main water supply always running; household water not stored; no secondary water source used; secondary water supply used was unimproved (according to JMP definitions [33]) or no answer provided; household water is not boiled. **Household hygiene domain:** household does not own food garden; human faeces not used on food garden. **Household socioeconomic domain:** no people aged <5 years in household/no answer; no people aged 5–17 years in household/no answer; no people aged ≥65 years in household/no answer; socioeconomic quintile 5 (wealthiest). **Village domain:** village rubbish disposed of in bushes only. **qPCR domain:** no *N*. *americanus* infection; no *Ancylostoma* infection; no *G*. *duodenalis* infection. **Environmental domain:** acidic soil pH; other soil types (clay, clay-loam, sandy-clay, variable); other landcover types (cropland/natural vegetation, evergreen forest, savanna).

## Discussion

This analysis presented the first investigation of combined WASH, environmental and demographic factors for intensity of STH infection in Timor-Leste. Using PCR-derived intensity of infection categorisation, similar infection intensity profiles to previous epg-based profiles [25] were found for each of *Ascaris* and *N*. *americanus*, with the most intense *Ascaris* infections in children, declining intensity and prevalence in adulthood, and prevalence and intensity of *N*. *americanus* being high in both childhood and adulthood. For *N*. *americanus*, heavy-intensity infections occurred in older age groups, although at low proportions. Whilst current risk factor models were not separately analysed by age groups, these results are in agreement with previous findings of different age-specific risk factors for different STH species in the study area [18]. This highlights the potential role of exposure-related risk factors, although other factors, such as acquisition of some level of immunity, may play a role [25].

It has previously been hypothesised that sex and age associations with STH are strongly related to exposure-associated behaviours [26]. Females showed a highly significant, increasing gradient for risk of heavy *N*. *americanus* infection with increasing age. Although less significant, a gradient was also evident for moderate-intensity *N*. *americanus* infection. Whilst overall male sex in those aged one to five was not a significant risk factor for *N*. *americanus* infection intensity compared to females of this age group, there was again an observation of greater heavy-intensity infection in males aged 18 to 64 relative to males aged one to five. These observations suggest that there are additional age- and sex-related factors occurring. This may include age as an expected indicator of time-accumulation given STH do not multiply in the host [14] and the longevity of *N*. *americanus* [27]. Alternatively, there could be exposure behaviours in older adults (compared to children) that are important to identify as they may be amenable to modification. There could also be differences in host immunity, particularly at different ages. Increased animal and soil contact through agricultural activities represents a direct potential transmission pathway (particularly in males) that requires further exploration. Further investigation into the female-age group association with heavy-intensity infection need to be undertaken; this could reflect particular household-related practices undertaken by women but not men. Further activities, such as constructing daily activity diaries, would be valuable to enable further insights in this setting. Alternatively, the findings of different sex and age patterns may be indicative of other factors such as host genetics [26].

Mixed-effects multinomial models were used to investigate the statistical relationship between intensity of STH infection, and WASH and environmental risk factors, whilst accounting for heterogeneity within village and household random effects. The lack of autocorrelation identified in semivariograms after accounting for large-scale environmental trends indicates that environmental variables explained the majority of spatial correlation in the data [19]. In our adjusted models incorporating WASH, demographic and environmental variables, environmental variables were generally associated with the greatest ARRs for infection intensity for both *N*. *americanus* and *Ascaris* spp. Precipitation was associated with increased risk of *N*. *americanus* infection of any intensity, but not *Ascaris* intensity. It is important to note that the precipitation variable included in these analyses was derived from 50-year averaged data from the driest month [19]; it is not reflecting seasonality, which could have had an impact on *N*. *americanus* survival rates in the soil [17]. Seasonal fluctuations could affect transmission potential but are not considered likely to have a strong influence on infection patterns given the longevity of *N*. *americanus* in the human host [17].

High rainfall contributes to suitably moist conditions for eggs and larvae to survive in soil, including the propensity for *N*. *americanus* larvae to remain near the soil surface and thus be available for human infection [17], but for *Ascaris*, excess rainfall may have negative impacts, possibly because the eggs sink lower in the soil as rainfall drains away. Our analysis showed strong associations between sandy-loam soil and highly increased risks of *N*. *americanus* infection, yet conversely, no significance in adjusted models for *Ascaris* spp. Observational associations between hookworms and sandy soil have been reported since the early 1900s (reviewed in [17]). Significance of soil type and rainfall likely reflect an important difference in life cycles and transmission potential between these two STH. *N*. *americanus* survive in the external environment as motile ensheathed larvae, but *Ascaris* spp. are present as (non-motile) eggs. The interrelated features of large-particle “sandy” soil, which tends to be less dense, aids both larval motility and water draining during/after rainfall, being therefore more amenable to *N*. *americanus* larval survival [17,26] and subsequent transmission potential. *Ascaris* eggs, on the other hand, are more susceptible to extremes, being able to dessicate in dry soil and to retard development in extremely wet soils [28]; this supports the lack of association between *Ascaris* spp., sandy soil and precipitation in this analysis. These factors, plus the shorter developmental time to infectivity in the soil of *N*. *americanus* compared to *Ascaris* [16], may contribute to the considerably greater prevalence of *N*. *americanus*.

We have previously reported on a protective association observed between alkaline soil type and *Ascaris* infection [19]; in this current analysis there was some evidence of a gradient of increasing infection intensity, although numbers were low and this finding therefore requires verification. Other studies use soil acidity data in spatial analyses [29,30], with one study reporting associations between acidic soil and increased infection risk ([30]; although this study used categories of pH that were all considered acidic compared to our definitions which defined neutral soil type as pH 6.6 to 7.3 [31]). Generally, soil acidity information is still rarely collected, yet this is an important potential determinant that could vary with precipitation, and other ecological or land use factors. Further analysis of pH ranges in epidemiological studies will contribute to knowledge of the optimal conditions for survivability of these helminths.

Differences in motility and survivability also potentially explain the direct association between increased elevation and *Ascaris* intensity of infection, with downhill runoff and draining after rainfall potentially facilitating survivability (and hence transmission) of those *Ascaris* eggs that remain in soil at higher elevations (i.e. those that do not get washed away); it would be plausible that *Ascaris* eggs that are washed downhill may be washed into rivers and streams, or lie within saturated environments that are less conducive to development. For *N*. *americanus* this was an inverse relationship for heavy-intensity infection; the protective association seen from elevation may reflect lower temperatures at higher elevations (as temperature was not included in multivariable analyses due to its high correlation with elevation). Negative correlations between hookworms and elevation, and, less consistently, positive correlations between *A*. *lumbricoides* and elevation, have previously been reported (reviewed in [17]).

Given high STH prevalence, poverty and poor existing WASH infrastructure [18], and the large quantity of risk factors investigated, few WASH risk factors have emerged in these analyses. Homogeneously poor access to improved WASH resources in study communities would limit our ability to find major associations [18], and is the most likely explanation for this. No significant WASH risk factors for *Ascaris* intensity of infection were found in adjusted models. For *N*. *americanus*, the protective association with boiling water against moderate-intensity infection is slightly surprising. There is evidence that *N*. *americanus* larvae can survive and remain infective for several days in water (decreasing with duration of water exposure) [32]. Whilst there is negligible published evidence for *N*. *americanus* infection via ingestion, this finding points to faecal contamination of drinking water sources as a possible exposure pathway. Water supply effects were also not significantly associated with intensity of *N*. *americanus* infection in the expected direction, with different levels of risk between surface water and an unprotected spring; both of which are unimproved water sources [33]. This could possibly be due to location of communities downhill from springs (thus positioned for gravity-fed flow), whilst communities may be generally uphill from surface water. There may additionally be a greater tendency for people to remove footwear when going to surface water, compared to (potentially smaller) springs. Alternatively, this may reflect heavy *N*. *americanus* contamination in the vicinity of particular water sources in the study area. Self-reporting error or a misunderstanding of water source definitions used in our study are also possible explanations. The protective effect of shared piped water, but not other ‘improved’ sources such as tubewells, is of interest and may reflect a heightened level of hygiene awareness in situations where multiple households use the same source, or alternatively, high correlation between some other variable and this one (although confounding and collinearity were investigated). The general lack of WASH associations, particularly with levels of sanitation, is similar to results that we have reported previously for prevalence [18]; however it was not previously clear whether this was because prevalence models were age-stratified, which could have affected power to detect effects. Lack of WASH risk factors therefore most likely reflects homogeneously poor access to WASH infrastructure, with flow-on impacts on amenable hygiene behaviours, in these communities, or a true lack of association with STH in this district. Alternatively, with a multinomial outcome, analyses could have adequate power to detect only moderate-large associations (see limitations).

Prevalence of *N*. *americanus* has previously been reported to be significantly associated with low socioeconomic status in this study area [18]. As has previously been identified socioeconomic status in this community reflects relative poverty that was still measurable within a general setting of poverty [18] and it is interesting that, for *N*. *americanus*, slightly higher estimates of association were seen for socioeconomic strata in heavy-intensity relative to moderate-intensity infection. This highlights an advantage of investigating socioeconomic status in defined districts on high-resolution (i.e. village-level) scale as opposed to national scale; it has been previously reported that between- and within-village heterogeneity may limit the usefulness of socioeconomic proxies in aggregated large-scale analyses [28]. The greater level of detail from this multinomial model provides additional insight into the *N*. *americanus*-poverty relationship. An interesting protective effect was the presence in the household of preschool-aged children; possibly this reflects adoption of hygienic behaviours when there are young children to protect them from disease exposures. The finding of reduced risk of heavy infection in people who reported three or more bowel motions is not surprising given that diarrhoea causes dilutive effects on quantities of helminths [34]. Our finding that recent deworming was not significantly associated with infection in adjusted models may be due to self-report error, with possible confusion about medications received.

The risks associated with environmental variables have important implications for STH control. The high rainfall, mountainous, tropical environment combined with high levels of poverty, poor WASH infrastructure and behaviour, and the longevity of STH eggs and larvae survival in soil [16], provides a fertile environment for STH transmission in this district. This is a challenge for helminth control because environmental variables themselves are not modifiable. Despite this, awareness of high-risk factors can influence other activities, primarily hygiene- and sanitation-associated behaviours to manage environmental risks. This provides a strong justification for investment in WASH activities irrespective of their individual statistical significance in risk factor analyses, as this is an exposure-reduction pathway that can potentially be manipulated. As current evidence for hygiene behaviours on STH control is sparse (reviewed in [1,2]), further research on the hygiene behaviours that could have greatest impact in this scenario needs to be undertaken as a priority.

This analysis is an important contribution to an ongoing RCT that will assess the benefits of augmented albendazole with WASH for STH control in Manufahi District, Timor-Leste [21]. As well as a detailed understanding of baseline WASH infrastructure and behaviours upon which to benchmark trial-related improvements in WASH, the knowledge of environmental factors is an essential prerequisite for effective targeting of interventions.

### Limitations and strengths

This is an observational analysis and, as such, cause and effect cannot be determined. As has been noted previously [18] much of the WASH data collected involved self-report of infrastructure and behaviours. Presence, type and cleanliness of household and village latrines were verified by interviewer observation. Self-reporting is a frequently-encountered drawback of measuring WASH characteristics. Further, extensive heterogeneity in assessing WASH behaviours on STH outcomes makes assessment of WASH characteristics challenging [15,35]. An important research priority is to develop specific WASH measurement guidelines for STH control. Power calculations indicated power to detect low associations for *N*. *americanus* and moderate associations for *Ascaris* infection intensity in multinomial models.

There are particular strengths to this study. This is one of very few epidemiological investigations of risk factors for STH infection intensity; this is particularly important to assess for environmental factors, given the links to STH transmission dynamics and correlations with morbidity. In this paper a community-based risk analysis is presented that combines high-resolution environmental, WASH and demographic variables in adjusted models. Advanced statistical techniques have been used to adjust for multinomial intensity outcomes, dependency of observations, effects of poverty, and confounding from other measured variables. As with all analyses, there is the possibility of residual confounding from unmeasured factors. However this provides the most comprehensive assessment of STH risk factors that we have identified in any setting.

A further strength is the use of PCR; a highly sensitive and specific technique [20] that is increasingly used for STH diagnosis. PCR-derived intensity of infection categorisation is a recent development, and requires further validation in different epidemiological settings [23]. Notwithstanding the need for further refinement of cut-points, different risk factors for moderate and heavy-intensity STH infections were found in this study area, with some evidence of a scale of increasing risk for factors such as soil type. This contributes useful, and highly relevant, information on risk factors within these communities. Use of infection intensity to determine risk factor associations requires more investigation. In particular, use of prevalence alone could mask significant intensity-related associations. This may mean that key evidence for WASH benefits may be overlooked in epidemiological studies that use prevalence of infection as the outcome. The possibility that WASH significance may be underreported in this way has been inadequately explored.

### Conclusion

With intensity of STH infection as the outcome, a comprehensive risk analysis of environmental, WASH and demographic variables is presented for communities in Manufahi District, Timor-Leste. Strong risk associations with environmental variables were identified. However, generally few associations with WASH risk factors were evident. This raises the importance of accurate measurement of WASH, and the need for clear guidelines on measuring WASH epidemiological research. This result also has important implications for STH control activities. Even in the absence of WASH significance, WASH infrastructure and behavioural-related activities are the only identified mechanism that could reduce or prevent transmission in an environment of high STH transmission potential. In this setting, anthelmintic treatment alone will not interrupt STH transmission; this provides a strong justification for application of integrated STH control strategies in this district.

## Methods

### Ethical approval and consent

This analysis used baseline data from 18 communities in a cluster randomised controlled trial (RCT), supplemented with data from an additional six communities, in Manufahi District, Timor-Leste (Australian and New Zealand Clinical Trials Registry ACTRN12614000680662) [21]. STH have recently been reported as endemic in this community, with prevalence of *N*. *americanus* of 60% and *Ascaris* spp. of 24%, as detected by qPCR [18].

The University of Queensland Human Research Ethics Committee; the Australian National University Human Ethics Committee; the Timorese Ministry of Health Research and Ethics Committee; and the University of Melbourne Human Research Ethics Committee approved the study protocol. Participant informed consent processes included explaining the study purpose and methods, and obtaining signed consent from all adults and parents or guardians of children under 18 years [21]. Children aged less than 12 months were excluded [21].

### Study setting, design and collection of data

The RCT commenced in May 2012. Detail on the RCT design is provided in the trial protocol [21]. A baseline survey of 18 communities involved in the RCT, and six additional communities, was conducted between May 2012 and October 2013. All communities surveyed were rural, and agrarian occupations predominated. Manufahi District has terrain varying from flat coastal plains to relatively mountainous inland areas (with elevation exceeding 1100 metres in some communities). It is a tropical region, with very high average rainfall of 190cm [19] and a wet season extending for close to ten months of the year. The average annual temperature is 24.5°C [19].

A single stool sample per participant was collected and fixed in 5% potassium dichromate. Multiplex qPCR was used to analyse stool samples for the presence and intensity of STH infection. Details on the qPCR diagnostic method are provided elsewhere [20].

Village, household and individual level questionnaires encompassing a broad range of potential WASH and socioeconomic risk factors were administered by trained field workers [18,21]. Interviewer observation of household and village latrines, their type and cleanliness was undertaken; all other questions were self-reported. Data were collated and entered into a Microsoft Access database and extracted to STATA 13.0 (Stata Corporation, College Station, Texas) for error checking.

### Data analysis

Individual-level data were linked to questionnaire and parasitological outcomes and household GPS coordinates [18,21]. Principal component analysis was used to create a wealth index, based on ownership of household assets (animals, transport and appliances), house floor type, reported income, and presence of electricity [18,22]. Using eigenvalues above 1, four principal components were retained and used to produce a final wealth score which was categorised into quintiles of relative socioeconomic status [18].

Outcome variables were intensity of *N*. *americanus* and *Ascaris* infection, which were analysed separately. Intensity of infection was derived from qPCR DNA cycle threshold (Ct) values, and categorised into two groups: (i) heavy-intensity, and (ii) moderate- to light-intensity infection (hereafter called “moderate intensity”) using algorithms generated from seeding experiments to correlate Ct-values to eggs per gram of faeces (epg) equivalents. Full detail of this method is provided elsewhere [20,23]. Exposure variables were WASH variables from study questionnaires, grouped into domains of related variables (e.g. household sanitation; household water supply; household hygiene; household socioeconomic status), and environmental variables that were sourced separately. Environmental variables were selected for analysis based on reported prior relationships with STH development [17], and availability via open-access sources. Temperature, precipitation, elevation, soil texture, soil pH, landcover and vegetation data were selected for analysis (Table 4) and processed using the geographical information system ArcMap 10.3 (ESRI, Redlands, CA) [19]. Very few environmental analyses incorporate information on soil texture and soil pH; it has been possible to incorporate these variables due to soil surveys conducted in the study region between 1960 and 1965 [24]; soil type was not considered to have changed dramatically since that time. A range of environmental variables related to the above factors was produced according to long-term average data, seasonal periods, and spatial resolution [19], with household as the data point, and a 1 km buffer applied (whereby the median raster value within a 1 km radius of the household was used [19]). Quality checks and exploratory analyses were undertaken to determine the most suitable version of each variable for analysis. Separate assessment of spatial autocorrelation was undertaken using semivariograms of residuals from multivariable models of selected environmental variables, with household and village random effects [19]; no additional autocorrelation was identified [19]. The analysis of environmental covariates in this study was limited to risk factor investigation. Predictive risk maps for STH infection in Manufahi District are published separately [19].

**Table 4:** Environmental variables selected for analyses.

Variable	Source	Temporal resolution	Spatial resolution
Temperature (°C)* Mean temperature in coldest quarter (June-August)–*Ascaris* model Temperature range (maximum temperature in the hottest month—minimum temperature in coldest month)–*N*. *americanus* model	WorldClim†	Monthly average ambient temperature from 1950–2000	1000m
Precipitation (cm)* Mean precipitation in wettest quarter (December-February)–*Ascaris* model Precipitation in driest month (September)–*N*. *americanus* model	WorldClim†	Monthly average precipitation from 1950–2000	1000m
Slope (°)	ASTER on Terra satellite^‡^	GDEM, 2001	30m
Elevation per 100m*	ASTER on Terra satellite^‡^	GDEM, 2001	30m
Vegetation Average normalised difference vegetation index (NDVI)*	MODIS Terra satellite^#^	01/01/2012-31/01/2013	250m
Soil pH* pH in 3 categories: Acidic (pH 5.5–6.5), neutral (pH 6.6–7.3), alkaline (pH 7.4–8.4)^§^	O Solos De Timor survey^$^	1960’s	N/A
Soil texture* Soil texture in binary: sandy-loam soil compared to other soil types (clay, clay-loam, sandy-clay, variable)^¤^	O Solos De Timor survey^$^	1960’s	N/A
Landcover Landcover in binary: woody savanna compared to other landcover types (cropland/natural vegetation, evergreen forest, savanna)^¤–^*Ascaris* model Landcover in binary: woody savanna and evergreen forest compared to other landcover types (cropland/natural vegetation, savanna)^¤–^*N*. *americanus* model	MODIS Terra and Aqua satellites^	2012	500m

**Notes:** Environmental variables from (19). †WorldClim Version 1.4 (release 3), ^‡^ASTER Global Digital Elevation Model Version 2, ^#^MOD13Q1 Version 5, ^$^O Solos De Timor data available from Seeds of Life Timor, ^MCD12Q1 Version 5.1, ^¤^Determined for Wash for Worms site [19]. ^§^Classified according to United States Department of Agriculture classification system (19, 31). *Variables used a 1km buffer being the median value in 1km radius of household. ASTER, Advanced Spaceborne Thermal Emission and Reflection Radiometer; GDEM, global digital elevation model. Environmental variable selection for *Ascaris* and *N*. *americanus* models based on Akaike’s Information Criterion.

Variables were investigated for multicollinearity according to likely relationships determined from literature, using tetrachoric analysis and the STATA “collin” user written package, according to the type of variable. Temperature and elevation were collinear; each variable was analysed in separate univariable models and subsequent variable selection was based on lower Akaike’s Information Criterion (AIC), indicating better predictive performance of the model. Chi-squared tests were conducted to compare intensity of infection by age, sex and socioeconomic quintile. Using categorised intensity of infection as the outcome, univariable and multivariable mixed effects multinomial regression was undertaken, with household and village random effects to account for dependence of observations. Regression analyses were undertaken for *N*. *americanus* and *Ascaris* spp. separately.

Regression models were not age-stratified due to insufficient numbers for some combinations of outcome and explanatory variables. Univariable regression was undertaken for each risk factor, with inclusion of variables in multivariable regression if they had *P<*0.2 on the Wald test in univariable analyses. All multivariable models included age group, sex, and socioeconomic quintile as covariates. Forward stepwise variable addition was used with variables retained if *P*<0.1 within, then across, domains of variables, until the most parsimonious adjusted model for each outcome was achieved. A categorised age variable, and a sex*age interaction term, were investigated, as the association between sex and the outcome was anticipated to vary by age group. Interactions were investigated by developing models without, then with, the interaction term and comparing these using the likelihood ratio test, with *P*<0.1 being the inclusion criterion for the interaction. Applying this criterion, the interaction term was retained in the final *N*. *americanus* model, but not the *Ascaris* model. A 5% significance level was used, however this analysis reports results of up to 10% significance, which is important for epidemiological interpretation. Analyses were conducted using generalised structural equation models in STATA 14.1 (Stata Corporation, College Station, Texas). Due to uncertainty regarding the linearity of the association of continuous environmental variables and the infection outcomes, quadratic terns were also investigated in all models; however as none of these quadratic terms were significant in the adjusted models, these results are not presented. Post-analysis power calculations indicated 80% power, with a 5% significance level, to detect relative risks of 1.2 to 1.8 for *N*. *americanus* infection intensity (depending on level of intensity), and, reflecting lower prevalence overall, relative risks of 2.7 to 3.9 for *Ascaris* infection intensity.

## Supporting information

S1 Checklist STROBE checklist for cross-sectional studies. (DOC)
